# Economic cost analysis of door‐to‐door community‐based distribution of HIV self‐test kits in Malawi, Zambia and Zimbabwe

**DOI:** 10.1002/jia2.25255

**Published:** 2019-03-25

**Authors:** Collin Mangenah, Lawrence Mwenge, Linda Sande, Nurilign Ahmed, Marc d'Elbée, Progress Chiwawa, Tariro Chigwenah, Sarah Kanema, Miriam N Mutseta, Mutinta Nalubamba, Richard Chilongosi, Pitchaya Indravudh, Euphemia L Sibanda, Melissa Neuman, Getrude Ncube, Jason J Ong, Owen Mugurungi, Karin Hatzold, Cheryl C Johnson, Helen Ayles, Elizabeth L Corbett, Frances M Cowan, Hendramoorthy Maheswaran, Fern Terris‐Prestholt

**Affiliations:** ^1^ Centre for Sexual Health HIV and AIDS Research Harare Zimbabwe; ^2^ Zambart Lusaka Zambia; ^3^ Malawi‐Liverpool‐Wellcome Trust Clinical Research Programme Blantyre Malawi; ^4^ Faculty of Public Health and Policy London School of Hygiene and Tropical Medicine London UK; ^5^ Population Services International Washington DC USA; ^6^ Society for Family Health Abuja Nigeria; ^7^ Liverpool School of Tropical Medicine Liverpool UK; ^8^ Ministry of Health and Child Care Harare Zimbabwe; ^9^ Faculty of Infectious and Tropical Diseases London School of Hygiene and Tropical Medicine London UK; ^10^ Department of HIV/AIDS World Health Organization Geneva Switzerland; ^11^ Institute of Psychology, Health and Society University of Liverpool Liverpool UK

**Keywords:** HIV self‐testing, costs and cost analysis, community, Malawi, Zambia, Zimbabwe

## Abstract

**Introduction:**

HIV self‐testing (HIVST) is recommended by the World Health Organization in addition to other testing modalities to increase uptake of HIV testing, particularly among harder‐to‐reach populations. This study provides the first empirical evidence of the costs of door‐to‐door community‐based HIVST distribution in Malawi, Zambia and Zimbabwe.

**Methods:**

HIVST kits were distributed door‐to‐door in 71 sites across Malawi, Zambia and Zimbabwe from June 2016 to May 2017. Programme expenditures, supplemented by on‐site observation and monitoring and evaluation data were used to estimate total economic and unit costs of HIVST distribution, by input and site. Inputs were categorized into start‐up, capital and recurrent costs. Sensitivity and scenario analyses were performed to assess the impact of key parameters on unit costs.

**Results:**

In total, 152,671, 103,589 and 93,459 HIVST kits were distributed in Malawi, Zambia and Zimbabwe over 12, 11 and 10 months respectively. Across these countries, 43% to 51% of HIVST kits were distributed to men. The average cost per HIVST kit distributed was US$8.15, US$16.42 and US$13.84 in Malawi, Zambia and Zimbabwe, respectively, with pronounced intersite variation within countries driven largely by site‐level fixed costs. Site‐level recurrent costs were 70% to 92% of full costs and 20% to 62% higher than routine HIV testing services (HTS) costs. Personnel costs contributed from 26% to 52% of total costs across countries reflecting differences in remuneration approaches and country GDP.

**Conclusions:**

These early door‐to‐door community HIVST distribution programmes show large potential, both for reaching untested populations and for substantial economies of scale as HIVST programmes scale‐up and mature. From a societal perspective, the costs of HIVST appear similar to conventional HTS, with the higher providers’ costs substantially offsetting user costs. Future approaches to minimizing cost and/or maximize testing coverage could include unpaid door‐to‐door community‐led distribution to reach end‐users and integrating HIVST into routine clinical services via direct or secondary distribution strategies with lower fixed costs.

## 1 Introduction

In East and Southern Africa, freely available HIV services have led to a 42% reduction in AIDS‐related deaths between 2010 and 2016. Despite such gains, 24% of people living with HIV (PLWH) remain undiagnosed 1. UNAIDS has set global targets for 90% of PLWH to know their status, 90% of known HIV‐positive individuals, to be on ART and 90% of those on anti‐retroviral therapy (ART) to have their viral load suppressed by 2020 2. To surpass and sustain high levels of awareness of HIV status, greater efforts are needed to ensure that HIV testing reaches those individuals who have not yet been tested for HIV. This, however, is likely to require more significant financial investments, innovative approaches and new technologies, including HIV self‐testing (HIVST).

HIVST is defined as a process where a person collects his/her own specimen (oral fluid or blood) and then performs an HIV test and interprets the result, often in a private setting, either alone or with someone they trust. The World Health Organization recommends HIVST to reach the “at risk” and “untested” populations including men as a complement to current conventional testing approaches, including facility‐based and targeted community outreach‐based testing 1, 3, 4, 5. The cost of HIVST kits has declined in some settings, with the OraQuick^®^ HIV self‐test now costing US$2 per kit in 50 low‐ and middle‐income countries 6. However, at US$2, it is around twice the price of standard HIV rapid diagnostic tests currently used for HIV testing in Africa 7. Although HIVST kit price may be higher, impact analyses show that it can have an important public health benefit and offer value for money if implemented as a complement to current testing approaches 4, 5.

The HIV Self‐Testing AfRica (STAR) project has delivered over one million HIVST kits in Malawi, Zambia and Zimbabwe between 2016 and 2017 through a combination of distribution approaches, including facility‐based distribution at outpatient departments, within voluntary medical male circumcision (VMMC) services and in the community. This study presents the costs of the model that uses community‐based distribution agents (CBDAs) to deliver HIVST either at people's homes or within the community setting, hereafter “the CBDA model,” to generate evidence to inform the scale‐up of cost‐effective HIV testing services (HTS).

## 2 Methods

### 2.1 Setting, intervention and evaluation

Table 1 presents key setting characteristics across countries. In short, the adult HIV prevalence rates in Malawi, Zambia and Zimbabwe were approximately 10.0%, 12.0% and 14.6% respectively 8, 9, 10. While Malawi and Zimbabwe CBDA model sites were exclusively rural, a third of Zambia sites were peri‐urban or urban. Malawian and Zambian distribution sites were fewer and each served large populations, while Zimbabwe delivered kits to a larger number of smaller communities. This difference in site size is also reflected in the unit costs of conventional facility‐based testing, with higher costs in the smaller facilities in Zimbabwe. It is also notable that men contribute only 26% to 37% of HTS clients in these facilities.

**Table 1:** Key setting characteristics

	Malawi	Zambia	Zimbabwe	Source
National HIV prevalence among adults 15 to 59 years (%)	10.0	12.0	14.6	8, 9, 10
Number of districts	4	4	8	11
Number of sites	11	16	44	11
Catchment population of sites: mean (range)	27,439 (5500 to 82,581)	18,266 (7673 to 50,094)	3196 (549 to 6699)	11
Location: rural (urban or peri‐urban)	11 (0)	16 (8)	44 (0)	11
Scale of current HTS – based on facility HTS in same communities and period	16,921	27,888	44,727	16
Men attendance at HTS – based on facility HTS – % men	34	37	26	8, 9, 10
Health facility HTS cost per person tested in US$: mean (range)	$5.03 ($2.96 to $9.24)	$4.24 ($2.49 to $6.24)	$8.79 ($3.38 to $21.51)	16

HTS, HIV testing services.

In the CBDA model, all individuals aged ≥16 years who were present in the homestead at the time of CBDAs’ home visit were eligible for self‐testing. Testing was done by the self‐tester themselves after kit use demonstration and information on test result interpretation and linkage to follow‐on care by the CBDAs. CBDAs provided a self‐referral card to all testers to facilitate linkage to the local health facility for confirmatory testing and care for individuals with reactive HIVST results. In some cases, CBDAs were present during the self‐test to provide reassurance and support if testers requested their presence or assistance. Table 2 presents the characteristics of the CBDA model implemented across countries. Narrative descriptions of the models can be found in Data S1. The impact of the CBDA model on uptake of HIV testing and ART is being evaluated in three cluster‐randomized trials (CRTs). Detailed methodology of these CRTs is published elsewhere 11.

**Table 2:** Overview of door‐to‐door community‐based HIVST delivery models

	Malawi	Zambia	Zimbabwe
Type of cadre used for distribution of HIVST kits	Trained CBDAs Some with prior experience distributing other reproductive health products for PSI	Trained facility and CBDAs Recruited from communities with prior links to respective health facilities	Trained CBDAs Information on HIVST and linkage to post‐test services
Mode of distribution	Door‐to‐door community‐based distribution PSI field teams‐maintained stocks	Door‐to‐door distribution by CBDA's within communities and households Facility‐based distributors‐maintained stocks for CBDAs	Campaign‐style door‐to‐door community distribution to households for four to six weeks PSI field teams‐maintained stocks
Services offered to HIV self‐test clients	Introduction and demonstration of HIVST kit use (including interpretation of results) CBDAs typically revisited clients a few days after dropping off the kit to: o enquire whether it had been used, o pick up the used kit o disclosed non‐reactive HIVST: referral to VMMC o disclosed reactive HIVST: referral to linkage to HIV care	Introduction and demonstration of HIVST kit use (including interpretation of results) CBDAs typically revisited clients a few days after dropping off the kit to: o enquire whether it had been used o pick up the used kit o disclosed non‐reactive HIVST: referral to VMMC o disclosed reactive HIVST: referral to linkage to HIV care	Introduction and demonstration of HIVST kit use (including interpretation of results) Follow‐on services by PSI‐Zimbabwe mobile outreach teams at one to two weeks post HIVST kit distribution o confirmatory HTS plus o family planning o blood pressure checks and CD4 count when available o clients alerted to linkages to government health facilities
Used HIVST kit returns	Specially designed and locked drop‐boxes to return used self‐test kits located: o at all intervention sites	Specially designed and locked drop‐boxes were used to return used self‐test kits, located: o at each facility and o local community public areas	Specially designed and locked drop‐boxes, located: o at CBDA's homestead o each health facility o local community public areas
CBDA reimbursement	Per HIVST kit distributed US$0.15 (MWK 100)	Monthly US$78 (ZMW 750) independent of performance. Later changed to: Per HIVST distributed US$0.52 (ZMW 5) and per used HIVST kit returned US$0.21 (ZMW 2)	Per ward campaign (four to six weeks) US$50 with a maximum of 100 kits per distributor Per HIVST client linking to any PSI outreach service: $0.20 in half of the evaluation clusters

HIVST, HIV self‐testing; CBDA, community‐based distribution agent; PSI, Population Services International; MWK, Malawi Kwacha; ZMW, Zambian Kwacha.

### 2.2 Costing methods

We estimated the full economic cost of delivering HIVST within the CBDA model from the providers perspective, following international costing guidelines 12. This included start‐up and training costs, prior to the first HIVST kit distributed. Annual costs were estimated, with implementation costs collected between June 2016 and May 2017, depending on country implementation timelines. Start‐up, training and all other capital costs were annualized using a 3% discount rate. All costs were converted to 2017 US dollars using average annual exchange rates and the dollar inflation rate 13, 14, 15.

This top‐down costing collated all financial expenditures and categorized each line item by input type and distribution model. Inputs were allocated to distribution sites following predefined allocation factors, based on project monitoring and evaluation (M&E) data, including the percentage of kits distributed, percentage of distributors based in each site, distance from central office and percentage of direct expenditures, which is a weighted average of the preceding allocation factors. Table S1 presents how each allocation factor was applied to input type. Further detail of the definitions of project phase and inputs can be found in Data S2.

To estimate economic costs, the expenditure analysis was complemented by a valuation of all other resources used in the CBDA model. Observations of distribution in each site strengthened the economists’ understanding of the intervention and allowed for collection of data on donated goods and services. As a vertical model, these were relatively limited, and include a value for district or health facility storage contributed by the public health system. During the life of the project, the price of HIVST kits dropped from nearly $4 per kit to $2 per kit. The latter was imputed in place of the higher observed prices as it was considered the relevant kit price for any decision‐making building upon this analysis. Total costs, total kits distributed and average cost per kit distributed were estimated at the country level, and for each country, at the site level. The latter provides a range of average costs by site and allows for identification of economies of scale.

### 2.3 Sensitivity analysis

We undertook a series of one‐way sensitivity analyses to assess the impact of key cost assumptions on the unit cost per HIVST kit distributed. We varied the discount rate used to annualize costs from the base case of 3% to 0% and 15% to capture the impact of not discounting or using a higher local central bank discount rate. Prevailing discount rates during the study period were 15% in Malawi, 12.5% in Zambia and 7% in Zimbabwe 13, 14, 15. We further evaluated the impact of applying alternative allocation factors that is swapping % of kits distributed and % of CBDAs per site. We varied annualization (economic life years) time frames: training & sensitization was varied between one and three years (base case is two years) and project start‐up life between 2.5 and 7.5 years (base case is five years) to assess impact if the project goes on for shorter or longer than assumed.

### 2.4 Scenario analysis

In anticipation of planned programme scale‐up by respective country ministries of health, we conducted scenario analysis varying salaries ±10% to assess the impact of integration into public health services, and variation in kit distribution by ±10%. We also modelled the impact of HIVST kit price between the observed average kit price (US$3.40), a recent Bill and Melinda Gates Foundation subsidized price (US$2) and a hypothetical price approximately equal to current rapid finger prick test price (US$1) 16. Finally, we estimated a best‐ and worst‐case scenario, the point where all the parameters yield the lowest/highest unit cost per kit distributed. To generate estimates that are comparable with the costs of ongoing facility HTS in the same communities in Malawi, Zambia and Zimbabwe 16, we also present costs without above site‐level costs and start‐up.

### 2.5 Ethics

The study did not involve patient‐level data collection; we did, however, obtain permission from ministries of health in the three countries to collate data from administrative, M&E records at facility level for cost allocation. Ethical approvals for the parent study were obtained from the Medical Research Council of Zimbabwe, Malawi College of Medicine Research Ethics Committee, University of Zambia Biomedical Research Ethics Committee, London School of Hygiene and Tropical Medicine Ethics Committee and University College London Ethics Committee. The trials are registered under the Clinical Trials Network (ClinicalTrials.gov) under registration numbers NCT02793804; NCT02718274; Pan African clinical trials registry PACTR201607001701788 for Malawi, Zambia and Zimbabwe.

## 3 Results

### 3.1 Community‐based distribution model programme outcomes

During the costing period, 152,671, 103,589 and 93,459 HIVST kits were distributed in Malawi, Zambia and Zimbabwe against the approximate targets of 62,500, 416,294 and 224,116 through a total of 138, 139 and 1009 CBDAs respectively. The average number of HIVST kits distributed was 12,538 (range: 4556 to 42,134) across 11 sites in Malawi, 7206 (range: 1758 to 20,450) across 16 sites in Zambia and 2124 (range: 319 to 4201) across 44 sites in Zimbabwe, where distribution was intentionally restricted by campaign duration (Table S2). Nearly half (49%, 51% and 43%, respectively) of the HIVST kits were distributed to men.

### 3.2 Total HIVST costs and cost composition

Table 3 summarizes the findings of the cost analysis. The total distribution costs were calculated as US$1,243,940.66, US$1,700,730.45 and US$1,293,135.00 in Malawi, Zambia and Zimbabwe respectively. Capital costs accounted for 3%, 4% and 2% of the total costs with start‐up costs accounting for 15%, 10% and 6% in Malawi, Zambia and Zimbabwe respectively. Within recurrent costs, personnel costs accounted for a significant portion of total costs, at 26%, 52% and 42% of costs in Malawi, Zambia and Zimbabwe respectively. Although the price of kits was centrally negotiated and thus the same across countries, kits contributed to the largest portion of total costs in Malawi (34%) and the second largest proportion in both Zambia and Zimbabwe (14% and 17% respectively).

**Table 3:** HIV self‐test kit distribution cost breakdown and key cost contributors (in 2017 US$)

Input type	Malawi Kits distributed: 152,671 12 months: June 2016 to May 2017		Zambia Kits distributed: 103,589 11 months: July 2016 to May 2017		Zimbabwe kits distributed: 93,459 10 months: August 2016 to May 2017	
	Intervention cost	%	Intervention cost	%	Intervention cost	%
Start‐up						
Training	$11,313.34	1%	$31,000.73	2%	$3,149.10	0%
Sensitization	$58,485.72	5%	$58,306.80	3%	$2,694.30	0%
Start‐up other	$108,409.87	9%	$84,745.15	5%	$75,942.83	6%
Capital costs						
Building and storage						
Central	$16,755.33	1%	$54,077.43	3%	$3,266.62	0%
Warehouse	$–	–	$–	–	$–	–
Site level	$–	–	$–	–	$–	–
Equipment						
Central equipment	$28,026.91	2%	$13,597.20	1%	$14,759.28	1%
Site level	$–	–	$–	–	$7,621.29	1%
Vehicles and bicycles	$3,162.38	0%	$–	–	$–	–
Other capital	$–	–	$–	–	$35.14	0%
Total costs (capital and start‐up)	$226,153	18%	$241,727	14%	$107,468	8%
Recurrent costs						
Personnel	$318,129.23	26%	$880,688.56	52%	$555,187.86	42%
HIV self‐test kits	$418,584.61	34%	$237,303.53	14%	$219,627.52	17%
Supplies						
T‐shirts, bags, flipcharts	$35,611.73	3%	$78,569.63	5%	$67,757.98	5%
Other supplies	$–	–	$–	–	$142,543.96	11%
Vehicle operation, maintenance and transport	$109,240.41	9%	$148,117.37	9%	$57,396.14	4%
Building operation/maintenance						
Central	$2,204.87	0%	$19,416.76	1%	$18,602.17	1%
Warehouse	$–	–	$–	–	$13,141.39	1%
Site level	$–	–	$–	–	$–	–
Recurrent training	$13,409.18	1%	$19,235.49	1%	$90,440.92	7%
Waste management	$–	–	$–	–	$554.89	0%
Other recurrent	$120,607.08	10%	$75,671.83	4%	$20,414.02	2%
Total costs (recurrent)	$1,017,787	82%	$1,459,003	86%	$1,185,667	92%
Total CBDA HIVST costs	$1,243,940	100%	$1,700,730	100%	$1,293,135	100%
Cost per kit distributed	$8.15		$16.42		$13.84	

Note that totals have been rounded to the nearest US$.

HIVST, HIV self‐testing; CBDA, community‐based distribution agent.

### 3.3 Unit costs

**Figure 1:**
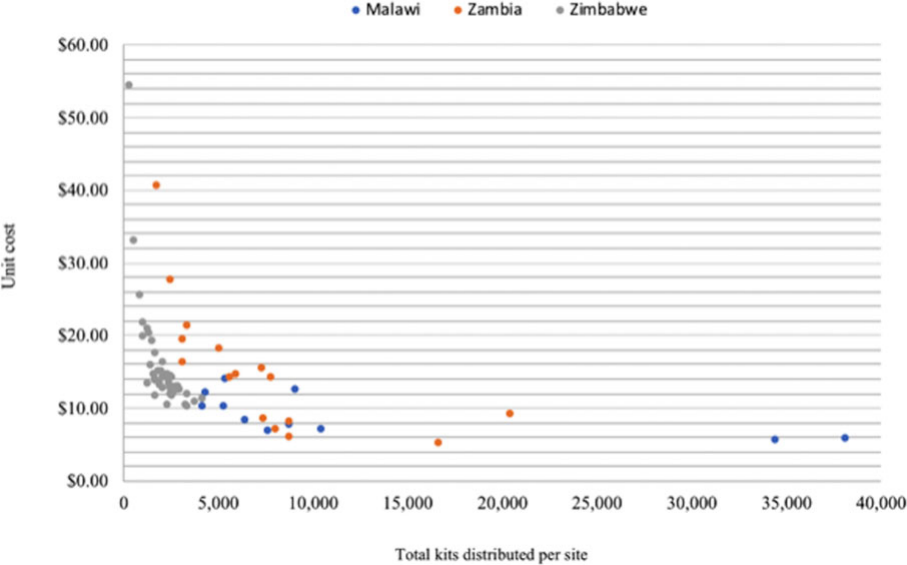
HIV self‐testing (HIVST) costs per HIVST kit distributed by site and quantity in 2017 US$.

The country‐level costs per HIVST kit distributed were US$8.15 for Malawi, US$16.42 for Zambia and US$13.84 in Zimbabwe. The cost per HIVST kit distributed across the sites ranged from US$7.20 to US$17.04 in Malawi, US$7.90 to U$50.00 in Zambia and from US$10.19 to US$54.44 in Zimbabwe. Figure 1 shows the unit cost per HIVST kit distributed plotted against the scale of HIVST kits across the three countries. Unit costs were generally lower at sites that were distributing a larger number of self‐test kits, suggesting a spreading of fixed costs across variable numbers of kits. When above site‐level and start‐up costs are removed our estimates were comparable to the facility HTS unit costs estimated in the same communities 16: US$6.67, US$10.42 and US$10.18 for the CBDA model, compared with facility HTS unit costs of $5.03 ($2.96 to $9.24), $4.24 ($2.49 to $6.24) and $8.79 ($3.38 to $21.51) in Malawi, Zambia and Zimbabwe respectively.

### 3.4 Sensitivity and scenario analysis

**Figure 2:**
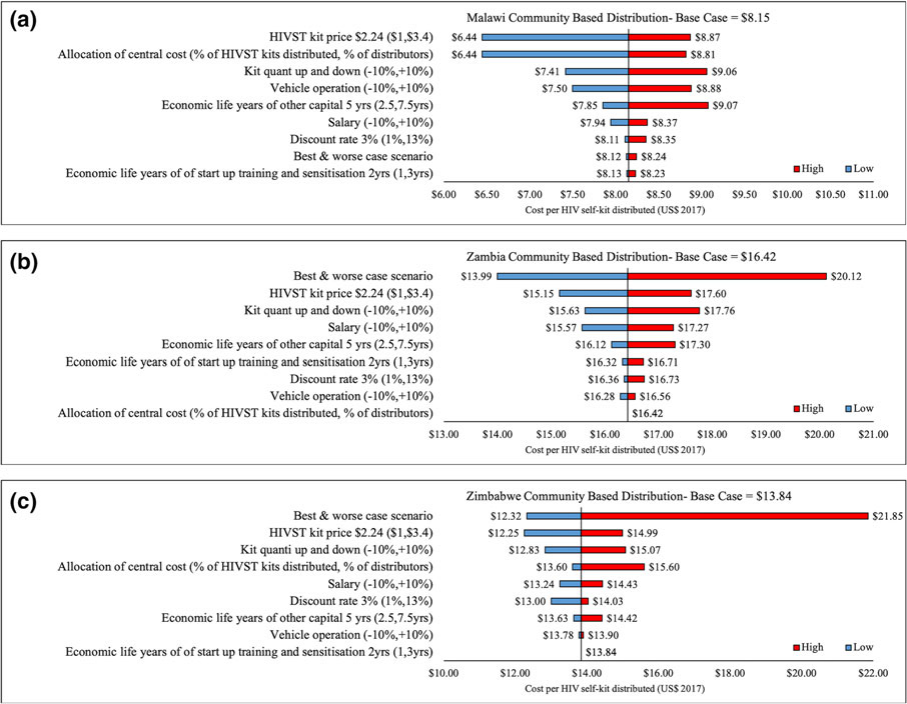
(a, b, c) Tornado diagrams of findings from deterministic sensitivity analysis (univariate and scenario analyses) in Malawi, Zambia and Zimbabwe.

Figures 2a,b,c show results from the univariate sensitivity and scenario analyses by country. Our unit costs per HIVST kit distributed remained robust when key cost parameters were varied. Varying life of start‐up training and sensitization between one and three years resulted in costs of US$7.85 and US$16.42 versus US$9.07 and US$15.05 in Malawi and Zambia respectively. For Zimbabwe, however, there was no change to the base case cost of US$13.84 as training and sensitization costs were classified as recurrent due to the sequential and short‐term nature of distribution across the eight districts, requiring training of CBDA who distribute for just four to six weeks. Varying life of start‐up life or development phase between 2.5 and 7.5 years resulted in costs of US$8.23, US$15.40 and US$14.42 compared to US$8.13, US$14.28 and US$13.63 in Malawi, Zambia and Zimbabwe respectively.

Varying HIVST kit price between US$1 and US$3.40 yielded costs of US$6.44, US$15.15 and US$12.25 versus US$8.87, US$17.60 and US$14.99 in Malawi, Zambia and Zimbabwe respectively. Varying salaries by ±10% yielded costs of US$7.94, US$15.57 and US$13.24 versus US$8.37, US$17.27 and US$14.43 respectively. Varying kit quantity by ±10% yielded costs of US$7.41, US$15.63 and US$12.83 versus US$9.06, US$17.60 and US$15.07 respectively. The best‐case scenario was US$6.14, US$13.99 and US$12.32 per kit distributed, whereas the worst‐case scenario was US$10.27, US$20.12 and US$21.85 per kit distributed.

## 4 Discussion

This is the first published study to present costs of door‐to‐door CBDA delivery of HIVST kits in Malawi, Zambia and Zimbabwe. Costs ranged from as low as US$7.20 at a very large distribution site where CBDA distribution of HIVST kits was integrated with the delivery of other health products, to US$54.55 with campaign‐style delivery in a very small community in Zimbabwe that would otherwise not have access to testing. Staff costs contributed a substantial portion of the costs highlighting potential opportunities for lower cost models from reconfiguring distribution to rely on unpaid volunteers within door‐to‐door community‐led distribution models. Additionally, economies of scale can clearly be optimized. In this analysis, we showed how unit costs fall as the number of kits distributed increases. As all modes of testing are scaled up and testing coverage increases, it will be critical to target populations efficiently, with special focus on communities underserved by facility‐based HTS.

Although costs are presented from a provider's perspective, door‐to‐door community HIVST distribution relieves users from substantial direct and indirect costs of attending health facilities. A study in these same communities in Malawi showed the mean costs of accessing HIV testing among women and men as US$1.83 and US$3.81, respectively, with men reporting significantly higher opportunity costs (i.e. lost income) 17. Community HIVST distribution reduces these costs to nearly zero, as kits are delivered in the home with no waiting times. We can, therefore, estimate the societal costs of facility‐based HIV testing in Malawi as US$6.86 for women and US$8.84 for men (the user costs reported above and the provider costs as reported by Mwenge et al. 16). This is comparable with our observed HIVST societal costs (excluding start‐up and above service level costs: US$6.67) in Malawi. Thus, HIVST may provide for unmet testing needs among remotely or never‐tested individuals, or others with high user costs of accessing facility‐based testing.

HIVST costs reflected across all three countries are not dissimilar to those reported previously in Malawi ($8.78 in 2016 US$) 18. We also found the cost of door‐to‐door community HIVST distribution to be comparable to standard community‐based HIV testing in sub‐Saharan Africa (range: US$7.37 to US$36.93) 19, 20. While we did find that CBDA delivered HIVST under this early demonstration and research programmes were more costly than facility‐based HIV testing 16, 18, we also found HIVST reached many more individuals. During the period of this costing study, health facilities serving the study communities provided HIV testing to approximately 17,000, 28,000 and 45,000 people, while the HIVST service distributed approximately 152,671, 104,000 and 94,000 kits in Malawi, Zambia and Zimbabwe respectively. Importantly, half of the HIVST kits were distributed to men, while only 26% to 37% of facility HIV testing clients were men 8, 9, 10, the population group primarily contributing to the HIV testing gap.

We anticipate potential for substantial economies of scale as HIVST programmes scale‐up and mature. The door‐to‐door community HIVST distribution model costed for this current study was implemented by a non‐governmental organization, under a research protocol, using paid and incentivized CBDAs and delivered to predominantly rural communities with no previous knowledge of, or experience with, HIVST. Interventions delivered in a research context tend to be associated with higher costs, as the primary objective is achieving effectiveness. Large‐scale implementation through door‐to‐door community‐led HIVST distribution with ordinarily paid government providers or community residents is likely to be significantly less costly. There are additional potential costs savings. First, we found costs were lower in high kit distribution sites suggesting economies of scale and ability to deliver at lower costs in more densely populated communities. Second, 10% to 20% of the costs were start‐up and initial capital costs, which would decrease as services mature. Third, as general populations and providers gain a better understanding of HIVST as a screening technology, we would expect less intense need for CBDAs (and therefore, less intense need for training workshops) and community sensitization activities.

Additionally, CBDAs could incorporate HIVST delivery into other health service activities thereby delivering cost savings to providers through economies of scope in services delivered by the CBDAs. Finally, as the HIVST market grows, technology advances and newer manufacturers enter, the price of HIVST kits will likely fall to prices comparable to blood‐based kits currently used in health facilities and in‐person support requirements could, in theory, could become cheaper than provider‐supervised testing. In this case, HIVST could save costs and allow providers to focus on confirmatory testing and strengthening linkage to ART 21, 22. To identify this, it will be important to take a full system costing approach. Such data have been collated and will be analysed jointly to inform cost‐effectiveness modelling.

From a research perspective, the wide cost variations highlight the importance of evaluating costs across a variety of settings in order to generate means and confidence intervals. Future analyses of these data may generate useful insights into efficiency and provide key inputs into modelled cost‐effectiveness analyses. It would also be important to expand conventional sensitivity analyses to assess unit costs when these observed ranges are included or when unit costs are incorporated as a function of scale. Furthermore, considering that our analysis only shows the costs of implementing CBDA model for a non‐governmental perspective and that these costs can vary if the kits were distributed differently, an important next research question will be to explore the costs of possible HIVST distribution modalities such as secondary distribution and social marketing models among others.

### 4.1 Limitations

The findings of our cost analyses are limited to unit costs per kit distributed as the private nature of the HIVST did not allow us to estimate the costs of identifying new HIV‐positive individuals or those HIV‐positive individuals linked to treatment through HIVST. In addition, our results are borne out of a research trial setting and may not truly reflect a real‐world situation: for example, site fixed transport costs are likely higher due to the distances between the trial communities, while in routine scale‐up, all communities would receive HIVST kits and transport would be shared across far higher scale.

Additionally, as HIVST was a new product, distribution was conservative, restricting the numbers of kits that each CBDA could distribute in Zimbabwe, and so constraining opportunities to operate at larger scale. Consequently, costs were likely higher than future routine implementation. The benefits of HIVST distribution may also be restricted by test performance characteristics such as sensitivity, specificity and ability of the user to read the test as well as rates of linkage to care. An important consideration would be the optimal, setting‐specific incentive structure for door‐to‐door community‐based distribution of the kits. It is important to highlight that for purposes of this analyses authors had not collated and analysed data on self‐test kit utilization. However, previous work has not only shown high uptake of HIVST but also high levels of kit utilization by recipients 4. Key strengths of this cost analysis are the estimation of costs across seventy‐one sites in three Southern African countries. The costing teams used standardized costing guidelines and collaboratively analysed data ensuring consistency of methods across countries and application of a range of sensitivity and scenario analyses exploring the impact of our assumptions.

### 4.2 Implications

Countries keen to achieve impact and meet the global testing and treatment targets will likely need to invest in a mixture of HIV testing approaches, including door‐to‐door community delivered HIVST targeted at populations with financial or other barriers to obtaining HIV testing in health services, that is people living in settings with high undiagnosed HIV or remote communities, and groups such as men and adolescents. Reducing costs during short‐term scale‐up and implementation of this model should focus on economies of scope and scale and ensure efficiencies in personnel and transportation costs. Alternative cost‐minimization approaches also need to be explored for acceptability, impact and affordability, aiming to provide affordable access to HIVST nationally, for example integrating HIVST within the existing facility and community health services, secondary distribution from facilities including partner delivered and peer‐network approaches.

## 5 Conclusions

Staff costs were a substantial cost contributor highlighting the potential for lower cost models if distribution relied on unpaid volunteers within door‐to‐door community‐led distribution models.

Economies of scale can also be optimized with our costs showing reductions when kits are distributed in higher numbers. Across all three countries, our HIVST cost estimates were not dissimilar to previous door‐to‐door community‐based HIVST and standard community‐based HIV testing models costed in sub‐Saharan Africa. Although the costs of CBDA delivered HIVST were higher than facility‐based HIV testing the evidence shows HIVST reaches many more individuals. A significant portion (almost half) of HIVST kits were distributed to men (key contributors to the HIV testing gap) compared to only 26% to 37% for facility HIV testing.

## Competing interests

The authors have no conflicts of interest to declare.

## Authors’ contributions

CM, LM, LS, NA, MD, HM and FTP conceptualized and designed the study. CM, LM, LS, NA, PC, TC and SK collected and facilitated the collection of data. CM, LM, LS, NA, PC, TC, SK, MD, JJO, HM and FTP analysed and interpreted the data. CM, LM, LS, NA, MD, PC, TC, SK, JJO, MM, MN, RC, PI, ELS, MNE, GN, OM, KH, CJ, HA, ELC, FC, HM and FTP drafted the manuscript and revised it critically. MM, MN, RC, PI, ELS, MNE, GN, OM, KH, CJ, HA, ELC, FC, HM and FTP supervised the study and facilitated the acquisition of the cost data. All co‐authors approved the final version to be published.

## Supporting information


**Table S1:** Cost allocation factors across the interventions by cost input type.
**Table S2:** Site‐level total and unit costs of HIVST and facility‐based testing.
**Data S1:** Narrative description of the CBDA models across countries.
**Data S2:** Definitions of cost category and cost inputs and allocation factors.
