# Supplementary material for: Economic cost analysis of door‐to‐door community‐based distribution of HIV self‐test kits in Malawi, Zambia and Zimbabwe
Source: J Int AIDS Soc. 2019 Mar 25;22(Suppl Suppl 1):e25255. doi: 10.1002/jia2.25255 (PMC6432106; doi:10.1002/jia2.25255)
Supplement: Supplementary file 1 — Table S1: Cost allocation factors across the interventions by cost input type. Table S2: Site‐level total and unit costs of HIVST and facility‐based testing. Data S1: Narrative description of the CBDA models across countries. Data S2: Definitions of cost category and cost inputs and allocation factors. [file JIA2-22-e25255-s001.docx]

**Supplement 1: Narrative description of the CBDA models across countries**

**Malawi**

In Malawi, the CRT was conducted in rural areas of Blantyre, Machinga, Mwanza and Neno districts in Southern Malawi and comprised a total trial population of approximately 62,500 residents. Catchment populations of 22 public rural primary health clinics (PHCs) were randomized 1:1 to either HIVST or standard of care. In the 11 HIVST intervention communities, residents had access to CBDA delivered HIVST over a continuous 1-year period (June 2016 to May 2017). CBDAs were paid an incentive of United States Dollar (US) $0.15 [100 Malawi Kwacha (MWK)] per kit distributed. This was integrated into their regular activities distributing contraceptives and other health products. In all sites, residents could access free HTS and ART if HIV-positive, through the PHCs.

**Zambia**

In Zambia, residents across 16 rural community sites had access to CBDA delivered HIVST over a continuous 1-year period (July 2016 to June 2017), reaching a total target adult population of 416,294 across Ndola, Kapiri, Lusaka and Choma districts. In this hub and spoke model CBDAs were linked to specific clinics and worked in their surrounding catchment populations. CBDAs were initially paid a monthly allowance of US$78 [750 Zambia Kwacha (ZMK)] independent of performance; this was later supplemented by a US$0.21 (2 ZMK) incentive per used kit returned. Though only six sites were included in the CRT, costs were evaluated for all 16 sites.

**Zimbabwe**

In Zimbabwe, the CRT was conducted across eight rural district sites with a total trial population of approximately 224,116 residents. Forty-four geographically defined wards were randomized 1:1 to either linkage intervention (HIVST plus distributor incentive for linkage events) or control (HIVST with fixed distributor allowance) clusters. HIVST was delivered across sites through one-off 4-6 week campaigns, moving sequentially from one district to the other between August 2016, and May 2017. In each district, new CBDAs were recruited and trained for three days. CBDAs then each distributed a specific number of tests proportional to their confined catchment area. Each CBDA was equipped with a tablet to demonstrate how to conduct a self-test through a video and to collate data on each self-tester.

At one to two weeks following HIVST distribution, the routine PSI mobile outreach service offered HIV confirmatory testing for individuals with reactive HIVST test result and HIV treatment referral to public sector health facilities for individuals with confirmed HIV positive results, including other services such as family planning and screening for non-communicable diseases. All CBDAs received a fixed allowance of USD$50, with an additional US$0.20 incentive for those in the linkage intervention arm per HIVST positive tester who linked for post-test services at PSI mobile outreach services. There was no compensation given to HIV negatives linking to post-test services. We estimated the cost of HIVST distribution in both intervention and –control sites. The cost of providing confirmatory testing at outreach services is not included in this study, for consistency across countries.

**Supplement 2: Definitions of cost category and cost inputs and allocation factors**

Start-up costs, including the costs incurred in providing training and sensitization activities, and all costs incurred during the period of intervention design and preparation to first distribution were treated as a capital cost as benefits of such investments would be expected to accrue to programmes over longer periods. Start-up costs were assumed to have a useful life of two years to reflect the lifespan of the STAR implementation.

Recurrent costs included the cost of personnel, HIVST kits and project operational activities which included vehicle operation costs such as fuel, insurance and maintenance for vehicles, building operations and maintenance, recurrent training, waste management costs and utilities. Building space included provider office space, warehouses and storage space at health facilities within distribution communities. Building operation and maintenance costs included rentals, utilities such as electricity and water, building insurance and security. Supplies included HIVST branded satchels, t-shirts, and hats, surge protectors, laptop bags, and power packs. Other supplies included office stationery such as bond paper, printer cartridges, first aid kits, envelopes, maps and pens, cellphone credit/airtime and internet data as well as utensils and office snacks and teas. Other recurrent costs included indirect expenses such as consultancies, office repairs and office fuel expenses.

**Table A1: Cost allocation factors across the interventions by cost input type**

| **Cost input type** | **Allocation factors to site level** | | | |
| --- | --- | --- | --- | --- |
|  | **Malawi** | **Zambia** | **Zimbabwe** |  |
| Training | % of distributors | % of distributors | % of distributors |  |
| Sensitization | % of communities within the site | % of direct expenditure | % of direct expenditure |  |
| Other Start-up | NA | % of HIVST kits distributed | % of HIVST kits distributed |  |
| Building and storage   - Central - Warehouse - Site level |  |  |  |  |
|  | % of direct expenditure | % of direct expenditure | % of direct expenditure |  |
|  | % of HIVST kits distributed | % of HIVST kits distributed | % of HIVST kits distributed |  |
|  | % of direct site level expenditure | % of direct site level expenditure | % of direct site level expenditure |  |
| Equipment   - Central equipment - Site level |  |  |  |  |
|  | % of direct expenditure | % of direct expenditure | % of direct expenditure |  |
|  | % of direct site level expenditure | % of direct site level expenditure | % of direct site level expenditure |  |
| Vehicles and bicycles |  | NA | NA |  |
| Other capital | % of HIVST kits distributed | % of HIVST kits distributed | % of HIVST kits distributed |  |
| Personnel | % of distributors | % of distributors | % of distributors |  |
| HIVST Kits | % of HIVST kits distributed | % of HIVST kits distributed | % of HIVST kits distributed |  |
| Supplies   - T-shirts, bags, flipcharts - Other supplies |  |  |  |  |
|  | % of HIVST kits distributed | % of distributors | % of distributors |  |
|  | % of HIVST kits distributed | % of HIVST kits distributed | % of HIVST kits distributed |  |
| Vehicle maintenance and transportation | % of mileage/distance (in km) | % of mileage/distance (in km) | % of mileage/distance (in km) |  |
| Building operations and maintenance   - Central - Warehouse - Site level |  |  |  |  |
|  | % of direct expenditure | % of direct expenditure | % of direct expenditure |  |
|  | % of HIVST kits distributed | % of HIVST kits distributed | % of HIVST kits distributed |  |
|  | % of direct site level expenditure | % of direct site level expenditure | % of direct site level expenditure |  |
| Waste management | NA | NA | % of HIVST kits returned |  |
| Other recurrent | % of HIVST kits distributed | % of HIVST kits distributed | % of HIVST kits distributed |  |

**Table A2: Site level Total & Unit costs of HIVST and Facility based testing**

| **Country** | **Site number** | | **Total HIVST kits distributed** | **Total intervention cost (Full)** | **Full Cost / kit distributed** | **Recurrent $ / pp tested*** | **Nearest facility HTS $ / pp tested (**[**16**](#_ENREF_16)**)** |
| --- | --- | --- | --- | --- | --- | --- | --- |
| Malawi |  | |  |  |  |  |  |
|  | 1 | | 9,329 | $89, 358.48 | $9.58 | $5.26 | $4.05 |
|  | 2 | | 4,556 | $53,387.87 | $11.72 | $5.72 | $3.86 |
|  | 3 | | 9,184 | $88,055.20 | $9.59 | $5.15 | - |
|  | 4 | | 7,731 | $66,691.09 | $8.63 | $4.76 | - |
|  | 5 | | 42,134 | $303,251.49 | $7.20 | $4.52 | $3.16 |
|  | 6 | | 29,941 | $231,897.62 | $7.75 | $4.61 | $4.68 |
|  | 7 | | 6,292 | $107,209.07 | $17.04 | $7.52 | $3.04 |
|  | 8 | | 9,922 | $133,192.52 | $13.42 | $6.81 | $2.96 |
|  | 9 | | 7,176 | $70,874.51 | $9.88 | $5.19 | $5.38 |
|  | 10 | | 4,608 | $61,093.47 | $13.26 | $6.81 | $5.81 |
|  | 11 | | 7,042 | $64,378.23 | $9.14 | $4.77 |  |
| Zambia |  | |  |  |  |  |  |
|  | 1 | | 5,587 | $105,822.48 | $18.9 | $11.61 | - |
|  | 2 | | 7,370 | $101,485.07 | $13.8 | $7.79 | - |
|  | 3 | | 3,113 | $81,341.94 | $26.1 | $15.71 | $6.15 |
|  | 4 | | 3,090 | $61,563.63 | $19.9 | $12.11 | $3.87 |
|  | 5 | | 20,450 | $161,774.90 | $7.9 | $6.40 | - |
|  | 6 | | 8,029 | $76,522.03 | $9.5 | $7.38 | - |
|  | 7 | | 8,759 | $93,243.83 | $10.6 | $8.40 | - |
|  | 8 | | 8,768 | $70,206.19 | $8.0 | $6.44 | - |
|  | 9 | | 7,752 | $158,721.75 | $20.5 | $10.17 | - |
|  | 10 | | 1,758 | $87,921.17 | $50.0 | $26.50 | $2.64 |
|  | 11 | | 5,030 | $130,696.73 | $26.0 | $13.36 | - |
|  | 12 | | 7,270 | $157,551.93 | $21.7 | $10.88 | - |
|  | 13 | | 4,902 | $116,784.17 | $23.8 | $13.62 | - |
|  | 14 | | 2,452 | $81,773.42 | $33.3 | $20.42 | $2.49 |
|  | 15 | | 5,895 | $121,294.01 | $20.6 | $11.70 | - |
|  | 16 | | 3,364 | $90,732.00 | $27.0 | $15.75 | $3.64 |
| Zimbabwe | |  |  |  |  |  |  |
|  | 22 | | 3,353 | $39,960.07 | $11.92 | $8.73 | $4.95 |
|  | 23 | | 2,891 | $36,484.04 | $12.62 | $9.27 | - |
|  | 24 | | 2,197 | $31,258.24 | $14.23 | $10.50 | - |
|  | 25 | | 1,966 | $29,505.61 | $15.01 | $11.09 | - |
|  | 26 | | 1,041 | $22,542.09 | $21.66 | $16.17 | $6.85 |
|  | 27 | | 578 | $19,065.95 | $32.98 | $24.81 | - |
|  | 28 | | 2,551 | $33,137.68 | $12.99 | $9.53 | $4.97 |
|  | 29 | | 2,123 | $30,787.85 | $14.50 | $10.71 | $10.49 |
|  | 30 | | 1,633 | $28,669.79 | $17.56 | $13.10 | $4.99 |
|  | 31 | | 2,941 | $37,059.67 | $12.60 | $9.25 | $3.87 |
|  | 32 | | 2,791 | $36,302.63 | $13.01 | $9.57 | $2.30 |
|  | 33 | | 4,201 | $46,958.62 | $11.18 | $8.16 | - |
|  | 34 | | 2,564 | $29,747.30 | $11.60 | $8.50 | $34.78 |
|  | 35 | | 1,646 | $22,668.12 | $13.77 | $10.17 | $4.56 |
|  | 36 | | 2,452 | $29,125.78 | $11.88 | $8.71 | $6.79 |
|  | 37 | | 2,616 | $31,896.61 | $12.19 | $8.98 | $2.74 |
|  | 38 | | 1,647 | $23,078.31 | $14.01 | $10.36 | $9.05 |
|  | 39 | | 1,931 | $25,845.10 | $13.38 | $9.89 | $8.90 |
|  | 40 | | 3,732 | $40,450.67 | $10.84 | $7.91 | $5.52 |
|  | 41 | | 1,403 | $22,161.10 | $15.80 | $11.71 | $3.01 |
|  | 42 | | 2,029 | $28,873.18 | $14.23 | $10.55 | $7.17 |
|  | 43 | | 1,806 | $27,092.58 | $15.00 | $11.15 | $2.54 |
|  | 44 | | 1,002 | $19,877.25 | $19.84 | $14.85 | $3.18 |
|  | 45 | | 2,489 | $31,729.73 | $12.75 | $9.39 | $3.90 |
|  | 46 | | 1,277 | $17,065.84 | $13.36 | $9.89 | $5.25 |
|  | 47 | | 3,388 | $34,511.24 | $10.19 | $7.44 | $4.15 |
|  | 48 | | 2,320 | $24,299.61 | $10.47 | $7.64 | $11.49 |
|  | 49 | | 1,696 | $19,847.36 | $11.70 | $8.59 | $5.11 |
|  | 50 | | 1,332 | $27,025.41 | $20.29 | $15.24 | $11.61 |
|  | 51 | | 1,485 | $28,640.85 | $19.29 | $14.47 | $12.41 |
|  | 52 | | 1,240 | $26,020.07 | $20.98 | $15.77 | $9.29 |
|  | 53 | | 2,030 | $33,007.35 | $16.26 | $12.12 | $13.94 |
|  | 54 | | 319 | $17,366.19 | $54.44 | $41.49 | $11.97 |
|  | 55 | | 893 | $22,739.56 | $25.46 | $19.21 | $20.93 |
|  | 56 | | 3,240 | $34,106.23 | $10.53 | $7.65 | $4.06 |
|  | 57 | | 2,521 | $30,200.13 | $11.98 | $8.79 | $4.06 |
|  | 58 | | 1,946 | $25,541.65 | $13.13 | $9.66 | $5.71 |
|  | 59 | | 2,353 | $31,612.65 | $13.44 | $9.96 | $4.28 |
|  | 60 | | 1,613 | $23,754.33 | $14.73 | $10.91 | $4.23 |
|  | 61 | | 2,071 | $26,507.47 | $12.80 | $9.41 | $4.08 |
|  | 62 | | 2,313 | $33,854.68 | $14.64 | $10.79 | $3.97 |
|  | 63 | | 2,448 | $35,059.27 | $14.32 | $10.56 | $4.17 |
|  | 64 | | 2,875 | $37,380.71 | $13.00 | $9.54 | $4.08 |
|  | 65 | | 2,516 | $35,870.54 | $14.26 | $10.51 | $4.22 |

* (Excludes start-up and training and above site level costs)
